# Oral Health‐Related Quality of Life After Corticobasal Implant‐Supported Rehabilitation: A Single‐Center Observational Study

**DOI:** 10.1155/ijod/8587233

**Published:** 2026-10-26

**Authors:** Fadia Awadalkreem, Nancy S. Farghal, Anita Gala Doshi, Md. Sofiqul Islam, Rasha Aziz Attia Salama, Haifa Kamal, Muhammed Mustahsen Rahman

**Affiliations:** ^1^ Department of Prosthodontics RAK College of Dental Sciences RAK Medical and Health Sciences University Ras Al Khaimah UAE; ^2^ Department of Dental Biomaterials Faculty of Dentistry Tanta University Tanta Egypt tanta.edu.eg; ^3^ Department of Prosthodontics Sankalchand Patel University Visnagar Gujarat India; ^4^ Operative Dentistry RAK College of Dental Sciences RAK Medical and Health Sciences University. Ras Al Khaimah UAE; ^5^ Community Medicine Department College of Medicine Ras Al Khaimah Medical and Health Sciences University Ras Al Khaimah UAE rakmhsu.ac.ae; ^6^ Community Medicine and Public Health Department Kasr El Ainy Faculty of Medicine Cairo University Giza Egypt cu.edu.eg; ^7^ Student Counselling and Lecturer, Psychology RAK Medical and Health Sciences University Ras Al Khaimah UAE; ^8^ Department of Periodontics RAK College of Dental Sciences RAK Medical and Health Sciences University Ras Al Khaimah UAE

**Keywords:** Corticobasal implant, GOHAI, implant treatment, oral health-related QoL, QoL, quality of life

## Abstract

**Objective:**

To evaluate changes in oral health‐related quality of life (OHRQoL) following Corticobasal implant‐supported prostheses, using the Geriatric Oral Health Assessment Index (GOHAI).

**Materials and Methods:**

This prospective, single‐arm pre‐/postobservational study included patients undergoing Corticobasal implant‐supported prosthetic rehabilitation at Narsinhbhai Patel Dental College and Hospital; no control or comparison group was included. Participants were recruited from January 2023 to August 2024. OHRQoL was assessed using the GOHAI at baseline and at the predefined 6‐month follow‐up visit. The final 6‐month assessment included in the analysis was completed in March 2025. Data were analyzed using SPSS Version 17.0. Paired *t*‐tests were used for within‐participant pre‐/postcomparisons, and repeated‐measures analysis of variance (ANOVA) was used to evaluate changes over time and exploratory differences in change across participant and treatment subgroups.

**Results:**

Of 116 enrolled participants, one was lost to follow‐up; 115 completed the 6‐month assessment and were included in the analysis (completion rate: 99.14%). Among them, 70.4% were female, 57.4% were aged ≥ 60 years, 78.3% were nonsmokers, and 57.4% had no reported medical history. The mean overall GOHAI score increased from 33.58 ± 7.36 at baseline to 50.84 ± 4.40 at 6 months, with a mean improvement of 17.26 points (95% CI: 15.77–18.75; *p* < 0.001; Cohen’s *dz* = 2.14). Significant improvements were observed in the functional, pain/discomfort, and psychological domains (all *p* < 0.001). Exploratory subgroup analyses suggested that the magnitude of GOHAI improvement may vary according to some participant and treatment characteristics; however, these findings were not adjusted for multiple comparisons and should be interpreted cautiously.

**Conclusion:**

Within the limitations of this prospective, single‐center observational study, OHRQoL, as assessed by the GOHAI, improved significantly from baseline to 6 months following Corticobasal implant‐supported prosthetic rehabilitation in the investigated cohort. These findings indicate favorable patient‐reported outcomes following Corticobasal implant‐supported prosthetic rehabilitation and should be interpreted in the context of the uncontrolled study design and the absence of a comparison group for evaluating implant‐system‐specific effects. Further controlled longitudinal studies with larger and more diverse populations are warranted to confirm these findings and evaluate their longer‐term clinical relevance.

## 1. Introduction

The World Health Organization conceptualizes health as encompassing physical, mental, and social well‐being, highlighting the importance of considering these interconnected dimensions when evaluating health‐related quality of life [1–4]. Oral health‐related quality of life (OHRQoL) reflects individuals’ perceptions of the impact of oral health on daily functioning and psychosocial well‐being [1, 5, 6]. Accordingly, assessment of dental treatment outcomes should extend beyond conventional clinical measures to include patient‐reported outcomes. Several instruments have been developed to assess OHRQoL, including the oral impact on daily performance [7], oral health impact profile (OHIP) [8–10], and geriatric oral health assessment index (GOHAI) [11–14]. The GOHAI, originally developed by Atchison and Dolan [11], is a validated 12‐item patient‐reported measure assessing physical function, psychosocial function, and pain or discomfort. It has been translated and validated in several languages, including Arabic, Chinese, Swedish, Hindi, Japanese, Malay, and French [15–21], supporting its use across diverse populations. The GOHAI has also been used to evaluate OHRQoL in patients undergoing prosthodontic and implant rehabilitation [9, 12–14] and was therefore selected for the present study.

Several methods have been introduced for the treatment of edentulism for improving esthetics, function, and QoL, which include removable, fixed, and implant‐supported prostheses [22]. Although dentures are conventionally used to replace missing teeth, some patients remain dissatisfied after denture insertion even when all clinical steps of denture construction are performed optimally [23]. Denture treatment success depends on various factors such as anatomical changes, adaptation period, denture‐base quality, and patient acceptance [24]. While evidence reported failure rates of 5%, 12%, and 41% at 2, 5–6, and >10 years, respectively [25], Shah et al. [26] highlighted a higher rate of dissatisfaction with mandibular dentures (36%) than with maxillary dentures (10%).

Currently, implant therapy is considered the gold standard treatment for partial and complete edentulism; it significantly improves the retention, support, and stability of the prostheses [27–30]. However, given the reduced bone quantity in patients with severely resorbed ridges, implant placement can be challenging and may require prior bone grafting [31–33], application of the all‐on‐four concept [31–33], or use of short [31–33] or basal implants [33–43].

Corticobasal implants represent a treatment option in clinical situations where conventional implant therapy may be challenging owing to inadequate alveolar bone volume or compromised trabecular bone. Reported clinical applications include the rehabilitation of severely resorbed ridges, immediate implant placement following tooth extraction, and the management of complex clinical conditions [33–43]. Implants are constructed from titanium or titanium alloy and designed to achieve mechanical anchorage through engagement of the cortical bone, which can provide high primary stability through osseofixation and facilitate immediate functional loading [33–43]. The bicortical screw implant (BCS) is a widely used Corticobasal implant with a polished smooth implant surface and one‐piece implant design [33–40]. The absence of an implant–abutment interface in a one‐piece implant design eliminates the potential of mechanical and biological complications associated with multicomponent implant designs [33–40]. Furthermore, the transfer of occlusal forces to the basal cortical bone contributes to favorable biomechanical performance during function [33–43]. In full‐arch rehabilitation, rigid splinting of individual implants with a metal framework further enhances load sharing and promotes more uniform biomechanical stress distribution across the prosthetic reconstruction [33–40]. However, available evidence supporting this treatment modality is derived from observational clinical studies, while comparative studies with conventional implant protocols remain relatively limited [33–40].

A systematic review by Awadalkreem et al. [36] reported survival rates ranging from 90.3% to 100% for intraoral basal implants, with BCS implants constituting the predominant implant design evaluated. Similarly, Lazarov [37] documented a 97.5% survival rate after 48 months of follow‐up. In a large clinical investigation involving 1019 BCS implants placed in patients with and without a history of periodontitis, Pałka and Lazarov [34] reported cumulative survival rates of 99.3%, 98.6%, and 97.0% at 12, 24, and 35 months, respectively. Likewise, Awadalkreem et al. [39] observed a 100% survival rate following placement of 171 BCS implants in patients presenting with compromised ridge conditions, with only limited complications reported. More recently, Doshi et al. [43] also reported a 100% implant survival rate after an 18‐month follow‐up of 380 implants and demonstrated a significant increase in peri‐implant bone density over the observation period. Collectively, these observational findings provide supportive evidence regarding the clinical performance and survival of Corticobasal implant systems, while implant survival should be considered separately from prosthetic and biological outcomes. Reported complications include prosthetic fractures, biomechanical overload, and technical complications associated with immediate loading [33–43]. Appropriate case selection, treatment planning, adherence to biomechanical principles, and regular clinical follow‐up remain important components of treatment.

Despite the encouraging clinical survival data, the effect of Corticobasal implant‐supported rehabilitation on OHRQoL remains less extensively investigated. Therefore, the present study aimed to evaluate changes in OHRQoL following Corticobasal implant‐supported rehabilitation using the GOHAI, a particular assessment of the functional, pain/discomfort, and psychosocial domains. Secondary exploratory analyses examined whether changes in OHRQoL differed according to sex, age, smoking status, implant location, restored jaw, and type of prosthetic rehabilitation.

## 2. Materials and Methods

### 2.1. Ethical Considerations

The study protocol was approved by the Ethics Committee of Narsinhbhai Patel Dental College and Hospital, Visnagar (NPDCH/IEC/2021/45). Written informed consent was obtained from each participant before enrollment. The study was reported in accordance with the strengthening the reporting of observational studies in epidemiology (STROBE) statement.

### 2.2. Study Sample

This prospective, single‐arm pre‐/postobservational study included patients undergoing Corticobasal implant‐supported prosthetic rehabilitation at Narsinhbhai Patel Dental College and Hospital. The participants were recruited from January 2023 to August 2024. OHRQoL was assessed at baseline before treatment and at the predefined 6‐month follow‐up visit, calculated from the date of prosthesis delivery. For the present study, only the baseline and 6‐month OHRQoL assessments were included in the analysis. The last participant included in the study underwent implant surgery on August 31, 2024 and received the prosthesis in September 2024. Accordingly, the final 6‐month follow‐up assessment included in the analyzed dataset was completed on March 1, 2025. Patients continued to undergo clinical follow‐up thereafter; however, observations beyond the 6‐month assessment were not included in the present analysis. Statistical analysis of the study dataset was performed in August 2025.

Based on clinic records and anticipated patient flow during the recruitment period, the accessible clinical population was expected to comprise ~165 patients undergoing Corticobasal implant‐supported prosthetic rehabilitation. Before recruitment, the target sample was estimated using the Raosoft sample‐size calculator [44], assuming a finite population of 165 patients, a 95% confidence level, a 5% margin of error, and a 50% response distribution, which yielded a target sample of 116 participants. This calculation was used for recruitment planning and was not an outcome‐based power calculation for the paired pre‐/post‐GOHAI analysis. No prospective outcome‐based power calculation was performed before recruitment, and no additional allowance for anticipated loss to follow‐up was incorporated into the prospective sample‐size estimate. During the predefined recruitment period, all consecutive patients were screened according to the eligibility criteria, and those who met the inclusion criteria were invited to participate after receiving a full explanation of the study protocol.

### 2.3. Inclusion and Exclusion Criteria

The inclusion criteria were as follows: adults (≥18 years) with partial or complete edentulism who underwent rehabilitation with Corticobasal implant‐supported prostheses because, following comprehensive clinical and radiographic evaluation, this treatment modality was considered the most appropriate treatment option based on the patient’s anatomical and clinical conditions. Patients for whom conventional endosseous implant therapy was considered the preferred treatment option were not included in the study.

All patients were treated using the BCS implant system (Dr. Ihde Dental AG, Gommiswald, Switzerland) and were classified as American Society of Anesthesiologists (ASA) physical status I or II and considered suitable candidates for implant surgery [45]. Participants also agreed to attend scheduled follow‐up visits [46] and provided written informed consent. The exclusion criteria included psychiatric or cognitive disorders; uncontrolled systemic diseases; active periodontal disease that could increase the risk of implant complications or failure; previous implant failure at the intended implant site; a history of radiotherapy or chemotherapy within the previous year [24, 25]; and treatment with conventional endosseous implants.

Eligibility for Corticobasal implant‐supported rehabilitation was assessed by the oral surgeon and implantologist following clinical and radiographic examinations. Treatment indications included inadequate alveolar bone volume or compromised ridge anatomy (available bone division C and D) that could limit conventional implant placement without bone augmentation [46], cases requiring immediate implant‐supported rehabilitation following extraction, and patients who declined bone‐grafting procedures and elected a nonaugmentation treatment approach [33, 39]. A total of 129 patients were screened for eligibility; six were excluded because their medical status was classified outside ASA Classes I–II. Among the participants classified as ASA II, the reported controlled systemic conditions included diabetes mellitus, hypertension, and asthma. Of the 123 eligible patients, seven did not proceed with enrollment: three declined informed consent, and four declined extraction of hopeless teeth required for treatment. A total of 116 participants were enrolled, one was lost to follow‐up, and 115 were included in the final analysis (Figure 1).

**Figure 1:**
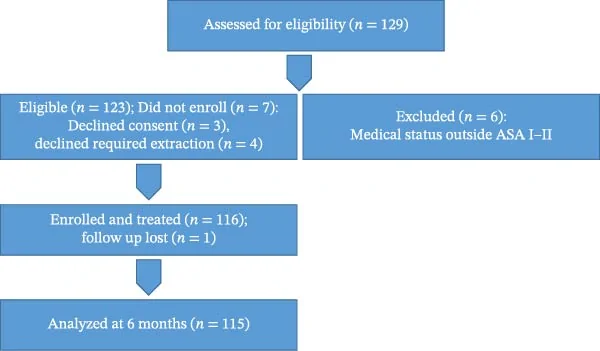
Participant flow through eligibility assessment, enrollment, treatment, 6‐month follow‐up, and analysis.

### 2.4. Surgical and Prosthetic Procedures

For standardization purposes, all patients were treated by the same maxillofacial surgeon and prosthodontist using a standardized surgical and prosthetic protocol (Figure 2A–I). First, preoperative clinical and radiographic images were obtained (Figure 2A–C). Subsequently, local infiltration anesthesia was administered (Lidocaine 2% with 1:100,000 concentration of epinephrine, Spain), followed by flapless implant osteotomy. The appropriate implant width and length were determined based on the available residual bone, implant location, and anatomical structures (Figure 2D–F). An impression was obtained using a monophase vinyl polysiloxane impression material (Ivoclar Vivadent AG, Schaan, Liechtenstein). As part of the standard postoperative protocol, all patients were prescribed amoxicillin (1 g twice daily for 5 days) and metronidazole (500 mg three times daily for 5 days). The first antibiotic dose was administered on the day of surgery following completion of the surgical procedure. Diclofenac potassium (Rapidus, 50 mg) was prescribed as needed for postoperative pain, up to three times daily, for a maximum of 3 days. The next day, the metal framework was constructed, and a try‐in was performed. The definitive restorations consisted of immediately loaded fixed implant‐supported prostheses with a metal framework veneered with composite resin and planned for replacement with ceramic restorations after 1 year. On the third day, the final prosthesis was cemented using Fuji Cement (GC Corporation, Tokyo, Japan), and occlusal adjustment was performed to establish a bilateral balanced occlusion. Patients were scheduled for follow‐up visits at 1 week, 3, 6, and 12 months, followed by annual visits afterwards. At each follow‐up visit, both clinical and radiographic examinations were performed (Figure 2G–I).

**Figure 2:**
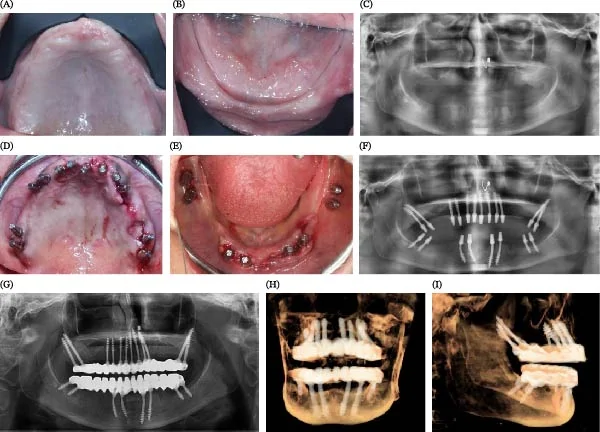
The clinical and radiographic presentation of the patient code 001. (A) Intraoral view of the patient showing maxillary edentulous arch. (B) Intraoral view of the patient showing mandibular edentulous arch. (C) The preoperative panoramic view of the patient. (D) The intraoral view of the maxillary jaw presenting implant distribution. (E) The intraoral view of the mandibular jaw presenting implant distribution. (F) The postimplant insertion panoramic radiograph showing the maxillary and the mandibular implant distribution. (G) The panoramic radiograph showing the maxillary and the mandibular implant‐supported prostheses. (H) The 3D cone beam CT showing the maxillary and the mandibular implant‐supported prostheses (frontal view). (I) The 3D cone beam CT showing the maxillary and the mandibular implant‐supported prostheses (lateral view). *Note:* Written informed consent for publication of the clinical and radiographic images was obtained from the patient.

During the 6‐month observation period, prosthetic complications were limited and manageable. Three patients experienced decementation of the prosthetic restoration, two of whom also required prosthetic adjustments. No implant loss, biological complications, surgical complications, or prosthetic fractures were recorded during the 6‐month follow‐up. No additional procedures beyond the reported prosthetic adjustments and management of decementation were required.

### 2.5. Questionnaire Design

The study questionnaire comprised four sections. The first section assessed the participants’ demographic and clinical characteristics. The second and third sections comprised the 12‐item GOHAI assessment administered at baseline before treatment and at the 6‐month follow‐up [14]. The fourth section recorded support patient‐reported information on satisfaction, pain, discomfort, mastication, appearance, phonetics, and patient‐reported complaints, where applicable.

The questionnaire was administered in English and was independently completed by 114 of the 115 participants included in the final analysis. One participant (0.87%) required Hindi‐language assistance. For this participant, a bilingual investigator who was not involved in treatment provided assistance using a written translated description to facilitate comprehension of the questionnaire items. The same written translated description and mode of administration were used at baseline and at the 6‐month follow‐up. This assisted administration did not represent administration of the formally validated Hindi version of GOHAI [18]. The validated Hindi GOHAI was not incorporated into the original study protocol because questionnaire administration had been planned in English, and only one participant subsequently required Hindi‐language assistance.

### 2.6. Assessment Tools

Sections 2 and 3 of the questionnaire comprised 12 items each across three domains: (1) functional activity (eating, speaking, and swallowing), (2) pain or discomfort, and (3) psychosocial effect [11]. Each item was scored on a five‐point Likert scale ranging from 1 to 5, yielding a total GOHAI score ranging from 12 to 60. The total GOHAI score was calculated as the sum of the 12‐item scores, with higher scores indicating better OHRQoL. The questionnaire was completed at baseline before treatment and at the 6‐month follow‐up.

As described by Atchison and Dolan [11], scores ≤50, 51–56, and 57–60 were considered low, average, and high, respectively. Higher total GOHAI scores indicated a better OHRQoL.

### 2.7. Questionnaire Validity and Reliability

The GOHAI is an established instrument with previously reported evidence of validity and reliability across multiple populations and languages [11–21]. In the present study, Cronbach’s alpha was 0.70 before treatment and 0.76 after treatment, indicating satisfactory internal consistency. Questionnaire validity was based on the established validation literature for the GOHAI.

### 2.8. Statistical Analysis

Data were analyzed using the Statistical Package for the Social Sciences (SPSS), with independent verification of all descriptive and inferential results against the original participant‐level dataset. Continuous variables were presented as mean ± standard deviation (SD) and categorical variables as frequencies and percentages. Analyses were based on complete cases; no GOHAI items were missing among the 115 participants completing the 6‐month follow‐up.

GOHAI total and domain scores were recalculated directly from the 12‐item‐level responses at baseline to the 6‐month follow‐up before analysis. The normality of the change scores and model residuals was assessed using graphical inspection and the Shapiro–Wilk test. Paired *t*‐tests were used for the baseline‐to‐6‐month comparisons. Cohen’s *dz*, calculated as the mean paired change divided by the SD of the paired change, was used to quantify paired effect sizes. For exploratory subgroup analyses, separate two‐time‐point mixed repeated‐measures analysis of variance (ANOVA) models were fitted for each subgroup variable (sex, age group, smoking status, anatomical region, restored jaw, and type of prosthetic rehabilitation), with time (baseline and 6‐month follow‐up) as the within‐participant factor and one subgroup variable at a time as the between‐participant factor. The subgroup *p*‐values reported in Table 1 correspond to the time × subgroup interactions and therefore test whether the magnitude of change differed across subgroup categories; they do not represent within‐subgroup baseline‐to‐6‐month *p*‐values. With only two‐time points, the assumption of sphericity is automatically satisfied. The homogeneity of variance of change scores was evaluated as part of the model diagnostics.

**Table 1:** Verified pre‐ and posttreatment GOHAI scores by subgroup.

Factor	Category	Overall GOHAI (before → after)	Function (before → after)	Pain/Discomfort (before → after)	Psychological (before → after)	Time × subgroup *p*
Sex	Male (*n* = 34)	34.41 ± 6.85 → 50.91 ± 3.85	10.97 ± 2.90 → 16.62 ± 1.91	8.97 ± 2.21 → 12.56 ± 1.85	14.47 ± 4.00 → 21.74 ± 1.48	Overall 0.514; Function 0.059; Pain 0.405; Psychological 0.636
	Female (*n* = 81)	33.23 ± 7.57 → 50.81 ± 4.64	9.79 ± 2.70 → 16.60 ± 2.04	9.41 ± 2.80 → 12.48 ± 1.24	14.04 ± 4.19 → 21.73 ± 2.04	
Age	≤39 (*n* = 16)	33.81 ± 8.17 → 47.69 ± 3.34	10.81 ± 3.97 → 15.62 ± 1.71	9.69 ± 2.87 → 11.38 ± 1.82	13.31 ± 4.96 → 20.69 ± 1.40	Overall 0.0038; Function 0.019; Pain 0.0085; Psychological 0.035
	40–59 (*n* = 33)	35.91 ± 9.22 → 50.58 ± 3.93	10.36 ± 2.98 → 16.45 ± 1.72	9.91 ± 3.08 → 12.48 ± 1.12	15.64 ± 4.75 → 21.64 ± 1.76	
	≥60 (*n* = 66)	32.36 ± 5.78 → 51.74 ± 4.53	9.86 ± 2.35 → 16.92 ± 2.12	8.86 ± 2.28 → 12.79 ± 1.35	13.64 ± 3.39 → 22.03 ± 1.97	
Smoking	Yes (*n* = 25)	31.64 ± 4.90 → 51.84 ± 3.92	10.08 ± 1.80 → 16.72 ± 2.07	8.60 ± 2.02 → 13.12 ± 1.01	12.96 ± 2.54 → 22.00 ± 1.80	Overall 0.0387; Function 0.753; Pain 0.0142; Psychological 0.0566
	No (*n* = 90)	34.12 ± 7.84 → 50.57 ± 4.51	10.16 ± 3.03 → 16.58 ± 1.98	9.47 ± 2.76 → 12.33 ± 1.49	14.50 ± 4.42 → 21.66 ± 1.91	
Region	Anterior (*n* = 12)	39.08 ± 6.26 → 48.83 ± 3.59	11.92 ± 3.82 → 16.75 ± 1.82	10.00 ± 2.45 → 10.75 ± 1.96	17.17 ± 4.75 → 21.33 ± 0.89	All *p* < 0.001
	Posterior (*n* = 30)	39.17 ± 8.22 → 51.20 ± 4.70	11.73 ± 3.68 → 16.80 ± 2.40	10.13 ± 2.50 → 12.57 ± 1.38	17.30 ± 4.46 → 21.83 ± 1.76	
	Both (*n* = 73)	30.38 ± 4.91 → 51.03 ± 4.37	9.19 ± 1.50 → 16.51 ± 1.86	8.81 ± 2.63 → 12.77 ± 1.15	12.38 ± 2.59 → 21.75 ± 2.05	
Jaw	Upper (*n* = 23)	36.91 ± 6.81 → 51.65 ± 4.63	12.17 ± 3.04 → 17.57 ± 2.02	9.22 ± 2.21 → 12.04 ± 1.66	15.52 ± 4.57 → 22.04 ± 1.61	Overall 0.0947; Function 0.150; Pain 0.518; Psychological 0.118
	Lower (*n* = 32)	34.22 ± 9.11 → 50.53 ± 4.45	9.84 ± 3.37 → 16.41 ± 1.95	9.66 ± 2.90 → 12.59 ± 1.43	14.72 ± 4.57 → 21.53 ± 2.05	
	Both (*n* = 60)	31.97 ± 6.04 → 50.70 ± 4.33	9.52 ± 1.92 → 16.35 ± 1.93	9.10 ± 2.65 → 12.63 ± 1.33	13.35 ± 3.55 → 21.72 ± 1.91	
Prosthesis	Partial (*n* = 35)	39.46 ± 7.35 → 49.63 ± 3.89	11.80 ± 3.66 → 16.54 ± 2.06	10.26 ± 2.57 → 11.71 ± 1.72	17.40 ± 4.53 → 21.37 ± 1.31	All *p* < 0.001
	Complete arch (*n* = 76)	30.80 ± 5.13 → 51.50 ± 4.53	9.36 ± 1.61 → 16.67 ± 1.93	8.80 ± 2.55 → 12.88 ± 1.10	12.64 ± 2.70 → 21.95 ± 2.10	
	Combination (*n* = 4)	35.00 ± 13.64 → 49.00 ± 4.40	10.50 ± 5.80 → 16.00 ± 2.94	9.75 ± 2.99 → 12.25 ± 1.89	14.75 ± 7.14 → 20.75 ± 0.96	Included in global interaction; no subgroup‐specific inference

*Note:* Values are mean ± SD. The *p*‐values shown represent global time × subgroup interaction tests and should not be interpreted as within‐subgroup baseline‐to‐6‐month *p*‐values. The combination rehabilitation group (*n* = 4) was included in the global exploratory three‐group time × prosthetic‐rehabilitation interaction model; however, no separate subgroup‐specific inferential test was performed for this group. Subgroup analyses were exploratory and were not adjusted for multiple comparisons. *p*‐values represent repeated‐measures ANOVA interaction tests assessing whether changes in GOHAI scores over time differed between subgroup categories.

Abbreviation: GOHAI, geriatric oral health assessment index.

Because the combination rehabilitation group contained only four participants, its subgroup‐specific values were presented descriptively, and no separate subgroup‐specific inferential testing was performed. The four participants were nevertheless retained in the global exploratory three‐group repeated‐measures model used to evaluate the time × prosthetic‐rehabilitation interaction. In the sensitivity analysis excluding the combination group, two‐sided independent‐samples *t*‐tests assuming equal variances were applied to participant‐level change scores (6‐month follow‐up minus baseline) to compare the partial‐arch and complete‐arch rehabilitation groups. Ninety‐five percent confidence intervals for the between‐group differences in mean change were calculated using the pooled‐variance standard error and the *t* distribution (*df* = 109).

All subgroup analyses were exploratory and were not adjusted for multiple comparisons. Accordingly, nominally significant subgroup findings were interpreted as hypothesis‐generating. Statistical significance for the prespecified overall baseline‐to‐6‐month analyses was set at *p* < 0.05; exact *p*‐values were reported where possible, and values smaller than 0.001 were reported as *p* < 0.001.

## 3. Results

### 3.1. Participants’ Characteristics

Of the 116 enrolled participants, one was lost to follow‐up, yielding a completion rate of 99.14%. The final analytic cohort therefore included 115 participants: 81 women (70.4%) and 34 men (29.6%). The mean age was 59.1 ± 13.8 years, and 66 participants (57.4%) were aged ≥60 years (Table 2).

**Table 2:** Participants’ characteristics, including participants’ gender, age, past medical history, smoking status, jaw to be restored, and region to be restored.

Variable	Number (*n*)	Percentage (%)
Gender		
Male	34	29.6
Female	81	70.4
Age (years)		
≤39	16	13.9
40–59	33	28.7
≥60	66	57.4
Reported medical history^a^		
Yes		
ASA I	31	27.0
ASA II	18	15.6
No	66	57.4
Smoking status		
Yes	25	21.7
No	90	78.3
Position (jaw to be restored)		
Upper	23	20
Lower	32	27.8
Both	60	52.2
Region to be restored		
Anterior	12	10.4
Posterior	30	26.1
Both	73	63.5
Prosthetic rehabilitation type		
Partial fixed implant‐supported prostheses	35	30.4
Complete arch fixed implant‐supported prostheses	76	66.1
Combination (one‐jaw complete/one‐jaw partial)	4	3.5

^a^Reported medical history refers to any documented previous or current medical condition, surgical history, or other relevant health‐related information recorded at the time of clinical assessment. Among participants classified as ASA I (*n* = 31), the recorded histories included seasonal allergies (*n* = 7), healed arm or leg fractures without residual impairment (*n* = 2), previous appendectomy with complete recovery (*n* = 3), previous uncomplicated dental surgery, including multiple tooth or wisdom tooth extractions (*n* = 10), and fully resolved urinary tract infections (*n* = 9). These participants had no active systemic disease affecting their ASA physical status at the time of assessment. Among participants classified as ASA II (*n* = 18), the recorded current controlled systemic conditions included controlled diabetes mellitus (*n* = 9), controlled hypertension (*n* = 6), and controlled asthma (*n* = 3).

Most participants were nonsmokers (*n* = 90, 78.3%). Of the 115 participants, 66 (57.4%) had no reported medical history, whereas 49 (42.6%) had a reported medical history. Among the total study population, 31 participants (27.0%) with a reported medical history were classified as ASA I. Their recorded histories comprised previous or resolved conditions without active systemic disease affecting their physical status at the time of assessment, including seasonal allergies, healed bone fractures, previous appendectomy, fully resolved urinary tract infections, and previous uncomplicated dental surgical procedures. The remaining 18 participants (15.6%) were classified as ASA II and had current controlled systemic conditions, including controlled diabetes mellitus, controlled hypertension, and asthma, without substantial functional limitation. Further details regarding the distribution of these conditions are presented in Table 2.

A total of 1610 implants were placed in 115 patients, corresponding to a mean of 14.0 implants per patient. Of these, 771 implants (47.9%) were placed in the maxilla and 839 (52.1%) in the mandible. In the maxilla, implant dimensions of 3.6 × 14, 3.6 × 17, and 3.6 × 20 mm were used in the anterior region, whereas dimensions of 3.6 × 20, 3.5 × 23, 3.6 × 26, 5.5 × 10, 5.5 × 12, and 5.5 × 14 mm were used in the posterior and pterygoid regions. In the mandible, implant dimensions of 3.6 × 17 and 3.6 × 20 mm were used in the anterior region, while dimensions of 3.6 × 12 and 3.6 × 14 mm were used posteriorly. Regarding treatment distribution, more than half of the participants (*n* = 60, 52.2%) received implant‐supported prosthetic rehabilitation in both jaws (Table 2). In addition, the majority of participants underwent rehabilitation involving both the anterior and posterior regions (*n* = 73, 63.5%).

Prosthetic rehabilitation was categorized into partial fixed implant‐supported prostheses, complete‐arch fixed implant‐supported prostheses, and combination rehabilitation (one‐jaw complete‐arch and the other partial‐arch fixed implant‐supported prosthetic). Most of the participants received complete‐arch fixed implant‐supported prostheses (*n* = 76, 66.1%) (Table 2).

As presented in Table 3, females predominated across all the prosthetic groups, particularly among complete‐arch fixed implant‐supported prostheses (72.4%) and combination rehabilitation (100%) groups.

**Table 3:** Distribution of demographic and clinical characteristics according to prosthetic‐rehabilitation type.

Variables	Partial fixed implant‐supported prostheses		Complete fixed implant‐supported prostheses		Combination	
	*N*	%	*N*	%	*N*	%
Gender						
Male	13	37.1	21	27.6	0	0.0
Female	22	62.9	55	72.4	4	100.0
Age (years)						
≤39	13	37.1	2	2.6	1	25.0
40–59	11	31.4	19	25.0	3	75.0
≥60	11	31.4	55	72.4	0	0.0
Position (jaw to be restored)						
Upper	16	45.7	7	9.2	0	0.0
Lower	9	25.7	22	28.9	0	0.0
Both	10	28.6	47	61.8	4	100.0
Region						
Anterior	11	31.4	1	1.3	0	0.0
Posterior	21	60.0	8	10.5	1	25.0
Both	3	8.6	67	88.2	3	75.0
Total	35		76		4	

Abbreviation: GOHAI, geriatric oral health assessment index.

Age distribution varied across prosthetic‐rehabilitation groups, with younger participants (18–39 years, 37.1%) with younger participants (18–39 years, 37.1%) more frequently represented in the partial‐arch fixed implant‐supported rehabilitation group, whereas older participants (age ≥60 years, 72.4%) were more prevalent in the complete‐arch fixed implant‐supported prostheses group (Table 3).

### 3.2. Overall GOHAI Scores Before and After Corticobasal Implant‐Supported Prosthetic Rehabilitation

Corticobasal implant therapy was associated with a significant improvement in the OHRQoL. The mean overall GOHAI score increased from 33.58 ± 7.36 at baseline to 50.84 ± 4.40 at 6‐month follow‐up. The mean paired improvement was 17.26 ± 8.06 points (95% CI: 15.77–18.75; *t* (114) = 22.97; *p* < 0.001; Cohen’s *dz* = 2.14). The functional domain increased from 10.14 ± 2.80 to 16.61 ± 1.99 (mean change 6.47; 95% CI: 5.91–7.03; *p* < 0.001; *dz* = 2.13), the pain/discomfort domain from 9.28 ± 2.63 to 12.50 ± 1.43 (mean change 3.23; 95% CI: 2.67–3.78; *p* < 0.001; *dz* = 1.07), and the psychological domain from 14.17 ± 4.13 to 21.73 ± 1.88 (mean change 7.57; 95% CI: 6.76–8.37; *p* < 0.001; *dz* = 1.73).

### 3.3. Association Between Sex and GOHAI Outcomes

The magnitude of overall GOHAI improvement did not differ significantly by sex (time‐by‐sex interaction *p* = 0.514). The interaction was also nonsignificant for pain/discomfort (*p* = 0.405) and psychological scores (*p* = 0.636), while the functional interaction approached but did not reach conventional statistical significance (*p* = 0.059). The pre‐/postvalues within each sex are presented descriptively in Table 1 and should not be interpreted using the interaction *p*‐values as within‐group tests (Table 1).

For the functional domain, scores in males and females increased from 10.97 ± 2.90 to 16.62 ± 1.91 and from 9.79 ± 2.70 to 16.60 ± 2.04, respectively. Similarly, pain/discomfort scores increased from 8.97 ± 2.21 to 12.56 ± 1.85 in males and from 9.41 ± 2.80 to 12.48 ± 1.24 in females, while psychological scores increased from 14.47 ± 4.00 to 21.74 ± 1.48 and from 14.04 ± 4.19 to 21.73 ± 2.04, respectively (Table 1). These within‐sex values are descriptive (Table 1).

### 3.4. Association Between Age and GOHAI Outcomes

Exploratory repeated‐measures analysis indicated that the magnitude of change differed across age groups for overall GOHAI (time‐by‐age interaction *p* = 0.0038), function (*p* = 0.019), pain/discomfort (*p* = 0.0085), and psychological scores (*p* = 0.035). However, these unadjusted interaction findings were exploratory and should be interpreted cautiously because multiple subgroup comparisons were performed.

Across the age groups, the ≥60‐year‐old group demonstrated the largest observed increases in both function and pain/discomfort scores. The significant time‐by‐age interaction for pain/discomfort (*p* = 0.0085) indicates that the magnitude of change differed across age groups and should not be interpreted as a within‐group baseline‐to‐6‐month significance test (Table 1).

### 3.5. Association Between Smoking and GOHAI

Smokers and nonsmokers both showed higher mean GOHAI scores at the 6‐month follow‐up than at baseline. The exploratory time‐by‐smoking interaction was significant for overall GOHAI (*p* = 0.0387) and pain/discomfort (*p* = 0.0142), but not for function (*p* = 0.753) or psychological scores (*p* = 0.0566). As these interaction analyses were unadjusted, the findings regarding smoking status and treatment response should be interpreted with caution.

### 3.6. Association Between Implant Location and GOHAI Outcomes

Implant location was analyzed according to both anatomical region (anterior, posterior, or combined) and the restored jaw (maxillary, mandibular, or both).

For anatomical region, the exploratory time‐by‐region interaction was significant for overall GOHAI (*p* < 0.001), function (*p* < 0.001), pain/discomfort (*p* < 0.001), and psychological scores (*p* < 0.001). Participants rehabilitated in both anterior and posterior regions had lower baseline scores and larger observed changes. Because these subgroup analyses were not adjusted for multiple comparisons and baseline scores differed substantially, these findings should be interpreted with caution.

For restored jaw, no statistically significant time‐by‐jaw interaction was detected for overall GOHAI (*p* = 0.0947), function (*p* = 0.150), pain/discomfort (*p* = 0.518), or psychological scores (*p* = 0.118). Pre‐/postvalues by the jaw group are presented descriptively in Table 1.

### 3.7. Association Between Prosthetic Rehabilitation Type and GOHAI Outcomes

Prosthetic rehabilitation received by the patients was categorized into partial‐arch fixed implant‐supported prostheses, complete‐arch fixed implant‐supported prostheses, and combination rehabilitation, where one jaw was replaced with a complete fixed implant‐supported prosthesis, while the other with a partial fixed implant‐supported prosthesis.

The *p*‐values in Table 1 refer to the global time‐by‐rehabilitation‐type interaction and not to within‐group pre‐/posttests. The combination rehabilitation subgroup (*n* = 4) was included in this global exploratory three‐group model; however, because of its very small sample size, no separate subgroup‐specific inferential testing was performed, and its subgroup‐specific values are presented descriptively.

The repeated‐measures analysis demonstrated significant overall effects of time for total GOHAI and all three domains (all *p* < 0.001). Partial eta‐squared (*ηp* ^2^) is reported in Table 4 as the effect‐size measure for the repeated‐measures ANOVA, whereas Cohen’s *dz* is reported separately for paired pre‐/postcomparisons.

**Table 4:** Verified repeated‐measures ANOVA for GOHAI total and domain scores, including time‐by‐prosthetic‐rehabilitation interaction effects.

Outcome	*F* time	*p* time	Partial *η* ^2^ time	*F* time × prosthesis	*p* interaction	Partial *η* ^2^ interaction
Overall GOHAI	815.95 (*df* = 1, 112)	<0.001	0.879	32.14 (*df* = 2, 112)	<0.001 ^∗∗^	0.365
Function	607.02 (*df* = 1, 112)	<0.001	0.844	10.25 (*df* = 2, 112)	<0.001 ^∗∗^	0.155
Pain/discomfort	155.65 (*df* = 1, 112)	<0.001	0.582	10.85 (*df* = 2, 112)	<0.001 ^∗∗^	0.162
Psychological	493.08 (*df* = 1, 112)	<0.001	0.815	25.89 (*df* = 2, 112)	<0.001 ^∗∗^	0.316

*Note:* Table 4 reports partial eta‐squared (*ηp* ^2^) for repeated‐measures ANOVA effects. The time effect has 1 and 112° of freedom, and the time × prosthetic‐rehabilitation interaction has 2 and 112° of freedom. Given the small size of the combination rehabilitation group (*n* = 4), the global interaction findings are considered exploratory; the combination subgroup was retained in the global model but was not subjected to separate subgroup‐specific inference, and a sensitivity analysis excluding it is reported in the Results section.  ^∗∗^ indicates statistical significance.

Significant time‐by‐prosthetic‐rehabilitation interactions were observed for total GOHAI (*F* (2,112) = 32.14, *p* < 0.001, *ηp* ^2^ = 0.365), function (*F* (2,112) = 10.25, *p* < 0.001, *ηp* ^2^ = 0.155), pain/discomfort (*F* (2,112) = 10.85, *p* < 0.001, *ηp* ^2^ = 0.162), and psychological scores (*F* (2,112) = 25.89, *p* < 0.001, *ηp* ^2^ = 0.316). Given the small number of participants in the combination rehabilitation group (*n* = 4), these interaction findings were considered exploratory and were supplemented by a sensitivity analysis excluding this subgroup.

In the sensitivity analysis, the mean change in total GOHAI was 10.17 points in the partial‐arch rehabilitation group and 20.70 points in the complete‐arch rehabilitation group, corresponding to a between‐group differences in change of 10.53 points (95% CI: 8.02–13.03; *p* < 0.001). Between‐group differences in change were also observed for the functional (2.57 points; 95% CI: 1.44–3.70; *p* < 0.001), pain/discomfort (2.62 points; 95% CI: 1.53–3.72; *p* < 0.001), and psychological domains (5.33 points; 95% CI: 3.89–6.77; *p* < 0.001). These posthoc exploratory sensitivity‐analysis findings should be interpreted cautiously because they may be influenced by baseline differences and residual confounding and should not be interpreted as demonstrating the superiority of one rehabilitation approach over another.

In the combined rehabilitation group (*n* = 4), the mean total GOHAI score increased from 35.00 ± 13.64 at baseline to 49.00 ± 4.40 at the 6‐month follow‐up. These values are presented descriptively because the small subgroup size precluded reliable subgroup‐specific inferential analysis.

## 4. Discussion

Immediately loaded Corticobasal implant‐supported rehabilitation has been increasingly reported as a treatment approach for patients with partial or complete edentulism, particularly in clinical situations involving reduced or compromised alveolar bone [33–43]. Previous observational studies have reported favorable clinical outcomes with Corticobasal implants, including implant survival rates ranging from ~96.8% to 100% across studies with different patient populations, treatment protocols, and follow‐up periods [34–41]. The Corticobasal approach is characterized by cortical bone engagement to achieve primary stability and facilitate immediate functional loading, and it has been used in selected clinical situations where bone augmentation might otherwise be considered, with the potential advantage of avoiding bone‐grafting procedures and their associated risks [33–43].

The present study evaluated changes in OHRQoL following Corticobasal implant‐supported prostheses as a treatment alternative for replacing missing teeth on patients’ OHRQoL using GOHAI. To our knowledge, the present study adds to the limited literature examining OHRQoL using the GOHAI among patients undergoing Corticobasal implant‐supported rehabilitation, underscoring the relevance of evaluating patient‐reported outcomes with this implant system. Corticobasal implant rehabilitation may offer the possibility of avoiding bone‐grafting procedures in selected patients with compromised ridge support and complex maxillofacial conditions, while previous studies have also reported satisfactory peri‐implant clinical outcomes [33–43].

The use of the GOHAI has been widely recommended as one of the best validated OHRQoL indicators worldwide by many investigators [11–21], justifying its selection in the present study as an accurate index assessing the patient’s OHRQoL and emphasizing the value of the present study.

The selection of the 6‐month time point for assessing the OHRQoL in the present study is grounded in biological and biomechanical clinical considerations because it represents a clinically stable stage after implant rehabilitation, during which early healing, neuromuscular adaptation, prosthetic accommodation, and functional recovery are generally established [14, 47, 48]. Earlier assessment may incompletely reflect transient postoperative discomfort and inadequate functional adaptation capacity, underscoring the complete benefits of the treatment [48]. This approach is supported by previous studies that reported significant improvement in the OHRQoL throughout the transition from implant placement to the definitive prosthetic‐rehabilitation phase [14, 47, 48]. Nevertheless, further longer‐term prospective studies are warranted to determine the sustained impact of Corticobasal implant rehabilitation on OHRQoL.

The higher proportion of females in this study is consistent with previous studies reporting sex‐related differences in dental service utilization and oral health behaviors [37, 49–53]. Evidence has identified significant sex‐related biological, behavioral, and sociocultural differences in oral health disease prevalence, seeking management, and prevention behavior, with women demonstrating higher oral health literacy and greater engagement in preventive dental care.

In the present cohort, the distribution of rehabilitation categories varied across age groups. This observation is consistent with previous epidemiological studies describing age‐related patterns of tooth loss, which may reflect the cumulative biological and functional burden of oral diseases over time [51, 54–56]. Evidence from India, the study area, indicates a substantial age‐related variation in the prevalence of edentulism, with a reported prevalence of 15.3% and individuals aged 50–59 years being 7.7 times more likely to be edentulous than those in older age categories [54]. Such age‐related patterns may be further influenced by access to dental care and preventive oral health practices, which collectively contribute to geographic variations in the prevalence of tooth loss [54–56].

The findings of the present study demonstrated a significant improvement in patient‐reported OHRQoL following rehabilitation with Corticobasal implant‐supported prostheses for the replacement of missing teeth. The mean GOHAI score increased by ~17 points, indicating an improvement in patient‐reported OHRQoL. This improvement is likely multifactorial and may be related, in part, to characteristics of the Corticobasal treatment approach, including cortical bone engagement to achieve high primary stability and facilitate immediate functional loading, with the potential to avoid bone augmentation in selected clinical situations [33–43]. The early delivery of a fixed implant‐supported restoration may also facilitate the restoration of mastication, speech, comfort, and social confidence. Collectively, these characteristics of immediately loaded Corticobasal implant‐supported rehabilitation may have contributed to the observed improvements across the GOHAI domains; however, this interpretation should be considered in the context of the absence of a comparative group in the present study.

The favorable patient‐reported outcomes observed in the present study are consistent with previous reports describing clinical and patient‐centered outcomes following Corticobasal implant rehabilitation [38–41]. Previous studies have reported favorable peri‐implant tissue health, high patient satisfaction, and significant improvements in oral function, esthetics, phonetics, and OHRQoL following treatment with Corticobasal implants [38–41]. Such outcomes have also been reported across diverse patient populations, including individuals with compromised ridge conditions and those presenting with systemic or local risk factors, including periodontitis, providing encouraging support for the clinical application of this treatment modality [38–41]. However, in the absence of a comparison group, the observed improvements may also reflect the broader benefits of receiving fixed implant‐supported prosthetic rehabilitation. Controlled comparative studies are therefore needed to further evaluate outcomes relative to other implant‐supported prosthetic‐rehabilitation approaches.

Moreover, the significant improvement in the OHRQoL observed in the present study is in accordance with previous reports of patient‐reported outcomes following other forms of implant‐supported rehabilitation [12, 14, 57–71]. In particular, Fillion et al. [14] reported significant improvements in OHRQoL, esthetics, masticatory function, and phonetics following fixed implant‐supported prosthetic rehabilitation, findings that closely align with the functional and psychosocial improvements observed in the present study and emphasized the positive outcomes associated with fixed implant therapy.

The observed improvement in the functional and psychological functions in a cohort of patients consisting predominately of completely edentulous patients who initially suffered from limited chewing ability can be related to the use of fixed implant‐supported prostheses, resulting in stable occlusal support and improved masticatory efficiency that enhance daily oral functions and patient confidence. A result that had been highlighted by many investigators [33–43] and supports the evidence showing the significant masticatory performance enhancement as well as the overall oral health quality of life with fixed implant‐supported prostheses compared to conventional complete dentures [13, 39, 40, 62]. Similarly, patients receiving complete‐arch rehabilitation may have experienced greater improvement because they generally presented with more extensive tooth loss and poorer baseline oral function, providing greater opportunity for improvement following rehabilitation rather than reflecting the treatment effect alone [14, 28, 47, 61]. A result is in line with many investigators [61–63]. However, the degree of quality improvement varies depending on the patient’s clinical and psychosocial characteristics. In contrast, Bramanti et al. [9] reported no significant influence of age, sex, or antagonistic teeth on patient satisfaction following implant treatment.

Pain/discomfort scores increased significantly from 9.28 ± 2.63 at baseline to 12.50 ± 1.43 at 6 months (mean paired change of 3.23 points; 95% CI: 2.67–3.78; *p* < 0.001; Cohen’s *dz* = 1.07), indicating improvement in this patient‐reported domain over the 6‐month follow‐up period. This finding is consistent with those of previous studies [34–41]. However, the absence of a control group precludes the causal interpretation of the observed improvement.

Both males and females demonstrated improvement in the OHRQoL with no significant sex‐related differences, which is in line with the findings of Mauland et al. [59], who reported 90% esthetic and functional satisfaction following implant treatment, with no sex variation. In contrast, Abbas Fedik and Diab [60] reported a significant influence of age and sex, with women being more aware of their oral health and appearance than men, while Awadalkreem et al. [38] documented significantly higher male satisfaction. These discrepancies may reflect differences in patient characteristics, baseline treatment expectations, cultural influences, and the study rather than the implant treatment itself.

Considering age variations, the result of the present study revealed significant improvement in oral health, especially among older patients. This finding may be attributed to the fact that the majority of participants were older adults, which is supported by the characteristics of the cohort. This result is consistent with the findings by Pjetursson et al. [61], who reported a positive impact of using posterior implant‐ and tooth‐supported prostheses, especially among older patients. While Gerritsen et al. [62] documented a significant impairment in the patient’s quality of life following teeth loss, Reissmann et al. [28] and Mora Rojas et al. [63] highlight the superiority of implant treatment over conventional dentures in complete edentulous patients’ rehabilitation; however, the degree of quality improvement varies depending on the patient’s clinical and psychosocial characteristics. In contrast, Bramanti et al. [9] found that satisfaction after implant treatment did not significantly vary based on age, sex, or antagonistic teeth (*p* > 0.05).

Both smokers and nonsmokers demonstrated improvement in GOHAI scores following Corticobasal implant‐supported rehabilitation. The unadjusted exploratory analysis showed significant time‐by‐smoking‐status interactions for overall GOHAI (*p* = 0.0387) and pain/discomfort (*p* = 0.0142), indicating differences in the magnitude of improvement over time between smokers and nonsmokers for these outcomes. No significant interactions were observed for the functional (*p* = 0.753) or psychological (*p* = 0.0566) domains. These findings should be interpreted cautiously because the subgroup analyses were exploratory and were not adjusted for multiple comparisons. Literature has reported variable findings regarding the influence of smoking on patient‐reported outcomes after Corticobasal implant treatment, with some studies reporting comparable patient satisfaction irrespective of smoking status, whereas others observed greater improvements among nonsmokers or higher satisfaction among smokers [40, 41]. These variations may reflect differences in study populations, baseline clinical characteristics, and follow‐up duration. Although smoking remains a recognized risk factor for peri‐implant complications and marginal bone loss, its influence on patient‐perceived OHRQoL appears to be less consistent [64–67].

The present study demonstrated a significant improvement in OHRQoL, irrespective of the implant position. This finding corroborates previous studies [68–71] that emphasize the significance of prosthesis design over implant position in affecting quality‐of‐life outcomes. In contrast, Bramanti et al. [9] and Yusa et al. [10] reported a significant improvement in patients’ reported outcomes following implant treatment in specific clinical situations, such as anterior implant rehabilitation or particular patterns of tooth loss. Kanehira et al. [13] linked the improvement of the GOHAI score to the number of occlusal supports replaced, irrespective of the position. However, these exploratory findings should be interpreted cautiously because subgroup differences may reflect differences in baseline GOHAI scores and treatment characteristics.

A strength of the present study is the prospective assessment of patient‐reported OHRQoL at predefined baseline and 6‐month follow‐up time points using the GOHAI in patients undergoing Corticobasal implant‐supported rehabilitation. The study contributes patient‐reported outcome data to an area in which such evidence remains limited. Furthermore, the structured analytical approach enabled systematic evaluation of overall and domain‐specific changes in OHRQoL, together with an exploratory assessment of differences across participant and treatment subgroups.

Certain limitations should be acknowledged; the study was conducted at a single center without a control group, which may limit generalizability and preclude causal inference. Potential sources of bias include selection bias, lack of blinding, response‐shift and social‐desirability effects, and regression to the mean. The cohort included both partially and completely edentulous patients, reflecting variation in baseline clinical and treatment characteristics.

One participant (0.87%) required Hindi‐language assistance using the same written translated description at baseline and follow‐up rather than the validated Hindi GOHAI [18]. Although the administration procedure was consistent at both assessment points and the assisting investigator was not involved in treatment, potential interviewer effects and linguistic measurement nonequivalence cannot be entirely excluded.

Subgroup analyses were exploratory and were not adjusted for multiple comparisons. In addition, subgroup sizes were unequal, particularly the combination rehabilitation group (*n* = 4), which was therefore considered primarily descriptively and further evaluated through sensitivity analysis excluding this group. Given the differences in baseline GOHAI scores and treatment characteristics between groups, subgroup findings should be interpreted cautiously and not as evidence of comparative treatment superiority. Future multicenter‐controlled longitudinal studies with larger samples, longer follow‐up, and complementary clinical and prosthetic outcomes would help further establish the generalizability and clinical relevance of these findings.

## 5. Conclusions

Within the limitations of this prospective, single‐center observational study, OHRQoL improved significantly from baseline to 6 months following Corticobasal implant‐supported rehabilitation, with improvements observed across the functional, pain/discomfort, and psychological domains within the investigated cohort. These findings indicate favorable patient‐reported outcomes following Corticobasal implant‐supported rehabilitation and should be interpreted within the context of the uncontrolled study design and lack of comparison with other implant systems for evaluating implant‐system‐specific effects. Further controlled longitudinal studies with larger and more diverse populations are warranted to confirm these findings and evaluate their longer‐term clinical relevance.

## Author Contributions

**Fadia Awadalkreem:** conceptualization, methodology, investigation, data curation, writing – original draft, writing – review and editing, validation, project administration. **Nancy S. Farghal:** conceptualization, writing – review and editing, validation. **Anita Gala Doshi:** conceptualization, investigation, data curation, validation, writing – review and editing. **Md. Sofiqul Islam:** conceptualization, writing – review and editing, validation. **Rasha Aziz Attia Salama:** formal analysis, data curation, validation, writing – review and editing. **Haifa Kamal:** conceptualization, writing – review and editing, validation. **Muhammed Mustahsen Rahman:** writing – review and editing, validation.

## Funding

This study did not receive any specific funding.

## Disclosure

All authors have read and approved the final version of the manuscript. Fadia Awadalkreem had full access to all of the data in this study and takes complete responsibility for the integrity of the data and the accuracy of the data analysis. All scientific work, analysis, and conclusions were independently conducted and verified by the authors.

## Ethics Statement

The study protocol was approved by the Ethics Committee of Narsinhbhai Patel Dental College and Hospital, Visnagar (NPDCH/IEC/2021/45). Written informed consent was obtained from each participant before enrollment.

## Conflicts of Interest

The authors declare no conflicts of interest.

## Data Availability

All data generated or analyzed during this study are included in this article.

## References

[1] Sischo L. and Broder H. L. , Oral Health-Related Quality of Life: What, Why, How, and Future Implications, Journal of Dental Research. (2011) 90, no. 11, 1264–1270, 10.1177/0022034511399918. PMC3318061 21422477

[2] Schramme T. , Health as Complete Well-Being: The WHO Definition and Beyond, Public Health Ethics. (2023) 16, no. 3, 210–218, 10.1093/phe/phad017. PMC10849326 38333767

[3] Yun S. , Ogawa N. , Izutsu M. , and Yuki M. , The Association Between Social Isolation and Oral Health of Community-Dwelling Older Adults: A Systematic Review, Japanese Journal of Nursing Science. (2023) 20, no. 3, 10.1111/jjns.12524, e12524. 36772867

[4] Hajek A. , Kretzler B. , and König H. H. , Oral Health, Loneliness and Social Isolation: A Systematic Review and Meta-Analysis, Journal of Nutrition, Health and Aging. (2022) 26, no. 7, 675–680, 10.1007/s12603-022-1806-8. PMC9166168 35842757

[5] Kovács S. D. , Suggestion for Determining Treatment Strategies in Dental Ethics, Journal of Bioethical Inquiry. (2024) 21, no. 2, 373–379, 10.1007/s11673-023-10310-2. PMC11289321 38032445

[6] Gagliardi D. I. , Slade G. D. , and Sanders A. E. , Impact of Dental Care on Oral Health-Related Quality of Life and Treatment Goals Among Elderly Adults, Australian Dental Journal. (2008) 53, no. 1, 26–33, 10.1111/j.1834-7819.2007.00005.x. 18304238

[7] Åstrøm A. N. , Haugejorden O. , Skaret E. , Trovik T. A. , and Klock K. S. , Oral Impacts on Daily Performance in Norwegian Adults: Validity, Reliability and Prevalence Estimates, European Journal of Oral Sciences. (2005) 113, no. 4, 289–296, 10.1111/j.1600-0722.2005.00225.x. 16048520

[8] Osta N. El , Haddad E. , Fakhouri J. , Saad R. , and Osta L. El , Comparison of Psychometric Properties of GOHAI, OHIP-14, and OHIP-EDENT as Measures of Oral Health in Complete Edentulous Patients Aged 60 Years and More, Quality of Life Research. (2021) 30, no. 4, 1199–1213, 10.1007/s11136-020-02709-w. 33226604

[9] Bramanti E. , Matacena G. , Cecchetti F. , Arcuri C. , and Cicciù M. , Oral Health-Related Quality of Life in Partially Edentulous Patients Before and After Implant Therapy: A 2-Year Longitudinal Study, Oral & Implantology. (2013) 6, no. 2, 37–42, 10.11138/orl/2013.6.2.037. PMC3808938 24175052

[10] Yusa K. , Ishikawa S. , and Suzuki N. , et al.Measures of Oral Health-Related Quality of Life in Patients With Bone Graft and Implant Prosthetic Rehabilitation at the Anterior of Mandible/Maxilla Among Young and Middle-Aged Adults: A Retrospective Pilot Study, International Journal of Implant Dentistry. (2023) 9, no. 1, 10.1186/s40729-023-00496-w. PMC10620345 37910231

[11] Atchison K. A. and Dolan T. A. , Development of the Geriatric Oral Health Assessment Index, Journal of Dental Education. (1990) 54, no. 11, 680–687, 10.1002/j.0022-0337.1990.54.11.tb02481.x. 2229624

[12] Nickenig H. J. , Wichmann M. , Terheyden H. , and Kreppel M. , Oral Health-Related Quality of Life and Implant Therapy: A Prospective Multicenter Study of Preoperative, Intermediate, and Posttreatment Assessment, Journal of Cranio-Maxillofacial Surgery. (2016) 44, no. 6, 753–757, 10.1016/j.jcms.2016.02.014. 27033149

[13] Kanehira Y. , Arai K. , Kanehira T. , Nagahisa K. , and Baba S. , Oral Health-Related Quality of Life in Patients With Implant Treatment, Journal of Advanced Prosthodontics. (2017) 9, no. 6, 476–481, 10.4047/jap.2017.9.6.476. PMC5741452 29279768

[14] Fillion M. , Aubazac D. , Bessadet M. , Allègre M. , and Nicolas E. , The Impact of Implant Treatment on Oral Health Related Quality of Life in a Private Dental Practice: A Prospective Cohort Study, Health and Quality of Life Outcomes. (2013) 11, no. 1, 10.1186/1477-7525-11-197. PMC3842837 24229381

[15] Atieh M. A. , Arabic Version of the Geriatric Oral Health Assessment Index, Gerodontology. (2008) 25, no. 1, 34–41, 10.1111/j.1741-2358.2007.00195.x. 18194334

[16] Wong M. C. , Liu J. K. , and Lo E. C. , Translation and Validation of the Chinese Version of GOHAI, Journal of Public Health Dentistry. (2002) 62, no. 2, 78–83, 10.1111/j.1752-7325.2002.tb03426.x. 11989210

[17] Hägglin C. , Berggren U. , and Lundgren J. , A Swedish Version of the GOHAI Index: Psychometric Properties and Validation, Swedish Dental Journal. (2005) 29, no. 3, 113–124. 16255355

[18] Mathur V. P. , Jain V. , Pillai R. S. , and Kalra S. , Translation and Validation of Hindi Version of Geriatric Oral Health Assessment Index, Gerodontology. (2016) 33, no. 1, 89–96, 10.1111/ger.12099. 24325659

[19] Naito M. , Suzukamo Y. , Nakayama T. , Hamajima N. , and Fukuhara S. , Linguistic Adaptation and Validation of the General Oral Health Assessment Index (GOHAI) in an Elderly Japanese Population, Journal of Public Health Dentistry. (2006) 66, no. 4, 273–275, 10.1111/j.1752-7325.2006.tb04081.x. 17225823

[20] Othman W. N. , Muttalib K. A. , and Bakri R. , et al.Validation of the Geriatric Oral Health Assessment Index (GOHAI) in the Malay Language, Journal of Public Health Dentistry. (2006) 66, no. 3, 199–204, 10.1111/j.1752-7325.2006.tb02580.x. 16913247

[21] Tubert-Jeannin S. , Riordan P. J. , Morel-Papernot A. , Porcheray S. , and Saby-Collet S. , Validation of an Oral Health Quality of Life Index (GOHAI) in France, Community Dentistry and Oral Epidemiology. (2003) 31, no. 4, 275–284, 10.1034/j.1600-0528.2003.t01-1-00006.x. 12846850

[22] Linn T. T. , Khaohoen A. , Thu K. M. , and Rungsiyakull P. , Oral Health-Related Quality of Life in Elderly Edentulous Patients With Full-Arch Rehabilitation Treatments: A Systematic Review, Journal of Clinical Medicine. (2024) 13, no. 12, 10.3390/jcm13123391, 3391. PMC11204181 38929921

[23] Yoshida M. , Sato Y. , Akagawa Y. , and Hiasa K. , Correlation Between Quality of Life and Denture Satisfaction in Elderly Complete Denture Wearers, International Journal of Prosthodontics. (2001) 14, no. 1, 77–80. 11842910

[24] Dutta S. R. , Passi D. , Singh P. , Atri M. , Mohan S. , and Sharma A. , Risks and Complications Associated With Dental Implant Failure: Critical Update, National Journal of Maxillofacial Surgery. (2020) 11, no. 1, 14–19, 10.4103/njms.NJMS_75_16. PMC7518499 33041571

[25] Moy P. K. , Medina D. , Shetty V. , and Aghaloo T. L. , Dental Implant Failure Rates and Associated Risk Factors, International Journal of Oral and Maxillofacial Implants. (2005) 20, no. 4, 569–577. 16161741

[26] Shah R. , Chaturvedi A. , Malek F. , and Prajapati D. H. , The Association Between Complete Denture Wearing and Denture Quality With Oral Health Related Quality of Life in an Elderly Gujarati Population, IOSR Journal of Dental and Medical Sciences. (2014) 13, no. 7, 30–38, 10.9790/0853-13713038.

[27] Sharka R. , Abed H. , and Hector M. , Oral Health-Related Quality of Life and Satisfaction of Edentulous Patients Using Conventional Complete Dentures and Implant-Retained Overdentures: An Umbrella Systematic Review, Gerodontology. (2019) 36, no. 3, 195–204, 10.1111/ger.12399. 30875108

[28] Reissmann D. R. , Dard M. , Lamprecht R. , Struppek J. , and Heydecke G. , Oral Health-Related Quality of Life in Subjects With Implant-Supported Prostheses: A Systematic Review, Journal of Dentistry. (2017) 65, 22–40, 10.1016/j.jdent.2017.08.003. 28789860

[29] Chakraborty N. , Almudarris B. A. , Gautam P. , Laddha R. , Giri T. K. , and Patel V. D. , Patient Satisfaction and Quality of Life Outcomes Following Dental Implant Placement, Journal of Pharmacy and Bioallied Sciences. (2024) 16, no. Suppl 4, S3338–S3340, 10.4103/jpbs.jpbs_831_24. PMC11805323 39926732

[30] Thieu M. K. L. , Mauland E. K. , and Verket A. , Satisfaction and Preferences Among Patients With Both Implant-Supported Single Crown and Tooth-Supported Fixed Dental Prosthesis: A Pilot Study, Acta Odontologica Scandinavica. (2023) 81, no. 5, 358–362, 10.1080/00016357.2022.2155239. 36519292

[31] Ali S. A. , Karthigeyan S. , and Deivanai M. , et al.Implant Rehabilitation for Atrophic Maxilla: A Review, Journal of Indian Prosthodontic Society. (2014) 14, no. 3, 196–207, 10.1007/s13191-014-0360-4. PMC4148518 25183902

[32] Soto-Penaloza D. , Zaragozí-Alonso R. , and Penarrocha-Diago M. , et al.The All-on-Four Treatment Concept: Systematic Review, Journal of Clinical and Experimental Dentistry. (2017) 9, no. 3, e474–e488, 10.4317/jced.53613. PMC5347302 28298995

[33] Ihde S. , Indications and Treatment Modalities With Corticobasal Jaw Implants, Annals of Maxillofacial Surgery. (2019) 9, no. 2, 379–386, 10.4103/ams.ams_142_19. PMC6933979 31909019

[34] Pałka Ł.R. and Lazarov A. , Immediately Loaded Bicortical Implants Inserted in Fresh Extraction and Healed Sites in Patients With and Without a History of Periodontal Disease, Annals of Maxillofacial Surgery. (2019) 9, no. 2, 371–378, 10.4103/ams.ams_147_19. PMC6933992 31909018

[35] Ahmad A. G. , Awadalkreem F. , and Osman M. , et al.Does the Protrusion of Corticobasal Implants in the Maxillary Sinuses Affect Sinus Health? A Retrospective Study, Journal of Contemporary Dental Practice. (2023) 24, no. 6, 357–363, 10.5005/jp-journals-10024-3521. 37534501

[36] Awadalkreem F. , Baroudi K. , Minervini G. , Zaineh Y. , and Al-Mehdhar E. , Basal Implants as a Treatment Alternative for Severely Resorbed Ridges, Minerva Dental and Oral Science. (2025) 74, no. 6, 393–404, 10.23736/S2724-6329.24.05066-6. 39841430

[37] Lazarov A. , Immediate Functional Loading: Results for the Concept of the Strategic Implant, Annals of Maxillofacial Surgery. (2019) 9, no. 1, 78–88, 10.4103/ams.ams_250_18. PMC6585197 31293933

[38] Awadalkreem F. , Khalifa N. , Satti A. , and Suleiman A. M. , The Influence of Immediately Loaded Basal Implant Treatment on Patient Satisfaction, International Journal of Dentistry. (2020) 2020, 10.1155/2020/6590202, 6590202. PMC7178505 32351569

[39] Awadalkreem F. , Khalifa N. , Satti A. , and Suliman A. M. , Rehabilitation of Patients With Compromised Ridge Support Using Immediately Loaded Corticobasal Implant-Supported Prostheses: A Prospective Observational Study, Journal of Contemporary Dental Practice. (2022) 23, no. 10, 971–978, 10.5005/jp-journals-10024-3416. 37073908

[40] Lazarov A. B. , The Impact of Diabetes, Smoking, and Periodontitis on Patients’ Oral Health-Related Quality of Life After Treatment With Corticobasal Implants: An Evaluative Study, Annals of Maxillofacial Surgery. (2021) 11, no. 2, 253–260, 10.4103/ams.ams_191_21. PMC8848706 35265494

[41] Sahoo S. K. , Mishra S. , Chinnannavar S. N. , Bajoria A. A. , Das A. , and Singh D. K. , Assessment of Oral Health-Related Quality of Life in Patients Receiving Corticobasal Dental Implants, Journal of Pharmacy and Bioallied Sciences. (2023) 15, no. Suppl 2, S1036–S1039, 10.4103/jpbs.jpbs_236_23. PMC10485463 37694072

[42] Gosai H. , Anchlia S. , Patel K. , Bhatt U. , Chaudhari P. , and Garg N. , Versatility of Basal Cortical Screw Implants With Immediate Functional Loading, Journal of Maxillofacial and Oral Surgery. (2022) 21, no. 3, 824–830, 10.1007/s12663-021-01638-6. PMC9474777 36274872

[43] Doshi A. , Patel J. , Gaur V. , Fernandes G. , and Awadalkreem F. , Assessment of the Peri-Implant Bone Density Following Bicortical Anchored Corticobasal Implants Placement in the Maxillary Arch: A Cross-Sectional Prospective Study, Journal of Contemporary Dental Practice. (2024) 25, no. 9, 820–829, 10.5005/jp-journals-10024-3755. 39791408

[44] Raosoft Inc. , Sample Size Calculator, 2004, http://www.raosoft.com/samplesize.html.

[45] Doyle D. J. , Hendrix J. M. , and Garmon E. H. , American Society of Anesthesiologists Classification, StatPearls, 2025, StatPearls Publishing. 28722969

[46] Misch C. E. , Contemporary Implant Dentistry, 2015, 4th edition, Mosby, 420–433.

[47] Manfredini M. , Pellegrini M. , and Rigoni M. , et al.Oral Health-Related Quality of Life in Implant-Supported Rehabilitations: A Prospective Single-Center Observational Cohort Study, BMC Oral Health. (2024) 24, no. 1, 10.1186/s12903-024-04265-y. PMC11069144 38704566

[48] Wen J. , Zhu W. , Han X. , and Wang X. , Impacts of Habit Formation Effect on Food Consumption and Nutrient Intake in Rural China, Nutrients. (2024) 16, no. 4, 10.3390/nu16040505. PMC10892789 38398829

[49] Kida I. A. , Åstrøm A. N. , Strand G. V. , and Masalu J. R. , Clinical and Socio-Behavioral Correlates of Tooth Loss: A Study of Older Adults in Tanzania, BMC Oral Health. (2006) 6, no. 1, 10.1186/1472-6831-6-5. PMC1471779 16536880

[50] Seman K. , Abdul Manaf H. , and Ismail A. R. , Association Between Functional Dentition With Inadequate Calorie Intake and Underweight in Elderly People Living in ‘Pondok’ in Kelantan, Archives of Orofacial Sciences. (2007) 2, 10–19.

[51] Khalifa N. , Allen P. F. , Abu-Bakr N. H. , and Abdel-Rahman M. E. , Factors Associated With Tooth Loss and Prosthodontic Status Among Sudanese Adults, Journal of Oral Science. (2012) 54, no. 4, 303–312, 10.2334/josnusd.54.303. 23221155

[52] Lukacs J. R. , Sex Differences in Dental Caries Experience: Clinical Evidence, Complex Etiology, Clinical Oral Investigations. (2011) 15, no. 5, 649–656, 10.1007/s00784-010-0445-3. 20652339

[53] Lipsky M. S. , Su S. , Crespo C. J. , and Hung M. , Men and Oral Health: A Review of Sex and Gender Differences, American Journal of Men’s Health. (2021) 15, no. 3, 10.1177/15579883211016361, 15579883211016361. PMC8127762 33993787

[54] Kailembo A. , Preet R. , and Stewart Williams J. , Common Risk Factors and Edentulism in Adults, Aged 50 Years and Over, in China, Ghana, India and South Africa: Results From the WHO Study on Global AGEing and Adult Health (SAGE), BMC Oral Health. (2016) 17, no. 1, 10.1186/s12903-016-0256-2. PMC4964081 27465011

[55] Borg-Bartolo R. , Roccuzzo A. , and Molinero-Mourelle P. , et al.Global Prevalence of Edentulism and Dental Caries in Middle-Aged and Elderly Persons: A Systematic Review and Meta-Analysis, Journal of Dentistry. (2022) 127, 10.1016/j.jdent.2022.104335, 104335. 36265526

[56] Schwendicke F. , Nitschke I. , Stark H. , Micheelis W. , and Jordan R. A. , Epidemiological Trends, Predictive Factors, and Projection of Tooth Loss in Germany 1997–2030: Part II. Edentulism in Seniors, Clinical Oral Investigations. (2020) 24, no. 11, 3997–4003, 10.1007/s00784-020-03265-w. PMC7544707 32246279

[57] Di Francesco F. , De Marco G. , and Capcha E. B. , et al.Patient Satisfaction and Survival of Maxillary Overdentures Supported by Four or Six Splinted Implants: A Systematic Review and Meta-Analysis, BMC Oral Health. (2021) 21, no. 1, 10.1186/s12903-021-01572-6. PMC8106178 33962612

[58] Nabil M. S. , Mahanna F. F. , and Said M. M. , Evaluation of Masticatory Performance and Patient Satisfaction for Conventional and 3D-Printed Implant Overdentures: A Randomized Crossover Study, BMC Oral Health. (2024) 24, no. 1, 10.1186/s12903-024-04389-1, 2672. PMC11162030 38851676

[59] Mauland E. K. , Bull V. H. , Melbye E. L. , and Verket A. , Patient-Reported Outcomes Following Dental Implant Rehabilitation According to Reason for Missing Teeth: A Norwegian Population Survey 8 Years After Treatment, Journal of Clinical Periodontology. (2024) 51, no. 2, 135–144, 10.1111/jcpe.13895. 37915235

[60] Fedik M. A. Abbas and Diab B. S. , Influence of Age and Gender on Oral Health Impact Profile Among Dental Implant Patients in Al-Karkh District in Baghdad, Iraq: A Cross-Sectional Study, Journal of the Pakistan Medical Association. (2024) 74, no. Suppl. 8, S259–S261, 10.47391/JPMA-BAGH-16-58. 39434317

[61] Pjetursson B. E. , Karoussis I. , Bürgin W. , Brägger U. , and Lang N. P. , Patients’ Satisfaction Following Implant Therapy: A 10-Year Prospective Cohort Study, Clinical Oral Implants Research. (2005) 16, no. 2, 185–193, 10.1111/j.1600-0501.2004.01094.x. 15777328

[62] Gerritsen A. E. , Allen P. F. , Witter D. J. , Bronkhorst E. M. , and Creugers N. H. J. , Tooth Loss and Oral Health-Related Quality of Life: A Systematic Review and Meta-Analysis, Health and Quality of Life Outcomes. (2010) 8, no. 1, 10.1186/1477-7525-8-126. PMC2992503 21050499

[63] Mora Rojas M. , Chauca Bajaña L. , Rodríguez Tates M. D. , Poussin L. , and Velásquez Ron B. , Oral Health-Related Quality of Life in Patients Rehabilitated With Dental Implants, Healthcare. (2025) 13, no. 7, 10.3390/healthcare13070813. PMC11989169 40218110

[64] Şahin T. , Evaluation of Implants in Smoking and Non-Smoking Patients With Peri-Implant Disease Risk Analysis and Esthetic Scores: An Observational Study, BMC Oral Health. (2023) 23, no. 1, 10.1186/s12903-023-03696-3. PMC10676582 38007513

[65] Windael S. , Vervaeke S. , De Buyser S. , De Bruyn H. , and Collaert B. , The Long-Term Effect of Smoking on 10 Years’ Survival and Success of Dental Implants: A Prospective Analysis of 453 Implants in a Non-University Setting, Journal of Clinical Medicine. (2020) 9, no. 4, 10.3390/jcm9041056, 1056. PMC7230390 32276371

[66] Alfadda S. A. , Current Evidence on Dental Implant Outcomes in Smokers and Nonsmokers: A Systematic Review and Meta-Analysis, Journal of Oral Implantology. (2018) 44, no. 5, 390–399, 10.1563/aaid-joi-D-17-00313. 29864381

[67] Hara M. , Matsumoto T. , Yokoyama S. , Higuchi D. , and Baba K. , Location of Implant-Retained Fixed Dentures Affects Oral Health-Related Quality of Life, Clinical Implant Dentistry and Related Research. (2017) 19, no. 4, 710–716, 10.1111/cid.12497. 28544590

[68] Annibali S. , Vestri A. R. , Pilotto A. , La Monaca G. , Di Carlo S. , and Cristalli M. P. , Patient Satisfaction With Oral Implant Rehabilitation: Evaluation of Responses to a Questionnaire, Annali di Stomatologia. (2010) 1, no. 3-4, 2–8. PMC3254380 22238708

[69] Emami E. , Heydecke G. , Rompré P. H. , de Grandmont P. , and Feine J. S. , Impact of Implant Support for Mandibular Dentures on Satisfaction, Oral and General Health-Related Quality of Life: A Meta-Analysis of Randomized Controlled Trials, Clinical Oral Implants Research. (2009) 20, no. 6, 533–544, 10.1111/j.1600-0501.2008.01693.x. 19515032

[70] Mustapha A. D. , Salame Z. , and Chrcanovic B. R. , Smoking and Dental Implants: A Systematic Review and Meta-Analysis, Medicina. (2022) 58, no. 1, 10.3390/medicina58010039. PMC8780868 35056347

[71] Lang L. A. , Hansen S. E. , Olvera N. , and Teich S. , A Comparison of Implant Complications and Failures Between the Maxilla and the Mandible, Journal of Prosthetic Dentistry. (2019) 121, no. 4, 611–617, 10.1016/j.prosdent.2018.08.002. 30600092

